# Three Siblings With Woodhouse-Sakati Syndrome: A Case Report of A New Saudi Family

**DOI:** 10.7759/cureus.32225

**Published:** 2022-12-05

**Authors:** Omar N Alhuzaim, Mohammad M Ahmad, Suphia M Sherbeeni, Fahad Almotawa, Abdulrahman S Ali, Abdul-Mohsen G Alhejaily

**Affiliations:** 1 Diabetes and Endocrinology, Obesity, Endocrine and Metabolism Center, King Fahad Medical City, Riyadh, SAU; 2 Endocrinology, Diabetes and Metabolism, Nera Medical Specialist & Day Surgery Center, Riyadh, SAU; 3 Internal Medicine, College of Medicine, University of Bisha, Riyadh, SAU; 4 Neurology, National Neuroscience Institute, King Fahad Medical City, Riyadh, SAU; 5 Oncology, Department of Basic Medical Science, Faculty of Medicine, Academic and Training Affairs, King Fahad Medical City, Riyadh, SAU

**Keywords:** hypogonadism, diabetes, autosomal recessive genetic disorder, omim: 80067, dcaf17, woodhouse-sakati syndrome

## Abstract

Woodhouse-Sakati syndrome (WSS) is a rare autosomal recessive multi-system genetic disease caused by loss of function mutations in the DCAF17 gene on chromosome 2q31.1. The disease is characterized by gradual neurologic degeneration and polyendocrinopathy, particularly noteworthy for hypogonadism, beginning in early adolescence. Clinical features show wide variability with no clear genotype-phenotype correlation. The pathophysiology of WSS is unclear at present and no specific treatment is available other than hormone replacement which is administered in the course of individualized symptomatic multidisciplinary care. Genetic testing helps in confirming the diagnosis along with genetic counseling of the patient and family members. Here we report multiple cases of WSS in three siblings from a new Saudi Arabia family who were diagnosed with WSS as a consequence of a common founder mutation in the DCAF17 gene with DNA analysis showing a homozygous single nucleotide frameshift deletion (c.436delC) in exon 4 of the gene.

## Introduction

Woodhouse-Sakati syndrome (WSS) is a rare autosomal recessive multisystem genetic disease predominantly affecting the neuroendocrine systems [1]. Neurologic features include progressive cognitive deterioration, extrapyramidal symptoms, sensorineural hearing loss, and MRI variances associated with leukoencephalopathy while endocrine manifestations include diabetes mellitus (T2DM), primary hypogonadism, and hypothyroidism. Various other features have also been described, including fast aging, retinopathy, alopecia, facial dysmorphism, and characteristic electrocardiography (ECG) changes [2]. Laboratory findings show decreased serum insulin-like growth factor 1 (IGF-1) and a hormonal profile consistent with hypogonadism [1]. The disease is caused by homozygous mutations in the DCAF17 gene (OMIM: 80067) located in chromosome 2q31.1, which encodes a nucleolar protein of unknown function. The majority of the DCAF17 gene mutations result in a protein that is unusually short, unstable, breaks down rapidly, or with compromised physiological function. The loss of this protein activity is believed to be responsible for the clinical features of WSS [3].

More than 30 isoforms of this protein exist, of which isoform alpha and beta are important and are highly conserved phylogenetically. The encoded protein is expressed ubiquitously at the nucleolar level. Overexpression of this protein in the brain, liver, skin, and gonads is observed in male mice, and its compatibility with neurological, cutaneous, and endocrine features of the disease further strengthens its role in pathogenesis [3]. WSS has no proven etiology yet, although it may be caused by dysfunctions that impact cellular aging or cell-cycle regulation. The defect in ribosome biosynthesis and other nucleolar processes may also be implicated as increased sensitivity to transcriptional blockades was observed in the nucleoli of lymphoblasts from patients with WSS [4, 5]. Many of the reported cases are believed to be due to a common familial mutation [6] and consanguinity has been proposed as a risk factor for WSS [7, 8]. It is believed that the variations in clinical features even within a single family could be caused by the impact of polymorphisms in other genes known as modifiers or modulators; however, no specific genes have been found so far to explain this phenomenon [3, 9].

## Case presentation

We are presenting the case of a female patient and her two brothers, all affected by WSS. They were born out of a consanguineous marriage between second cousins.

Patient 1

Our index case is a 23-year-old Saudi lady who presented to our endocrinology clinic for primary amenorrhea, along with primary hypothyroidism and T2DM. She was carried to full term with normal vaginal delivery and had an uneventful childhood with normal milestones, though her school performance was poor compared to her other normal siblings, leading her to drop out during secondary school. She first consulted a pediatrician and gynecologist around 10 years back at her local hospital for delayed puberty and primary amenorrhea. She then had a brief hormonal workup for which no further details are available, and had a prescription for conjugated estrogen and medroxyprogesterone. Seven years ago, she developed T2DM and primary hypothyroidism and was started on oral tablets of levothyroxine and metformin with sitagliptin.

On examination, she showed loss of scalp hair in the fronto-temporo-parietal distribution, which was progressive over the years, along with almost complete loss of eyebrows and eyelashes. She had a prominent forehead along with a long and triangular face. Her eyeballs were widely spaced and the nasal bridge was prominent. Secondary sexual characteristics were underdeveloped with Tanner stage 3 breasts and pubic hairs. There was no history of swallowing difficulty, seizures, abnormal limb posturing or movement, or any postural disfigurements. Visual, hearing, and dental assessments along with the rest of the examination were normal.

On investigation, high follicle-stimulating hormone (FSH) and luteinizing hormone (LH) along with low estradiol levels suggested hyper-gonadotropic hypogonadism. Thyroid function tests revealed primary hypothyroidism with normal anti-thyroid antibody titer. The glycated hemoglobin levels were 8.9%. Adrenal function tests were normal, along with a normal short synacthen test. She had low IGF-1 with normal IGF-binding protein-3 levels. Random screening growth hormone (GH) level was low and GH deficiency was confirmed with a clonidine stimulation test. Serum prolactin was within normal range. Vitamin D deficiency was also observed along with low normal serum-corrected calcium levels and secondary hyperparathyroidism. Her hemoglobin was 9.0 gm/dL along with low serum vitamin B12, which on further workup revealed a normal celiac profile with negative intrinsic factor assay, though her gastric parietal cell antibody titer was high.

**Figure 1:**
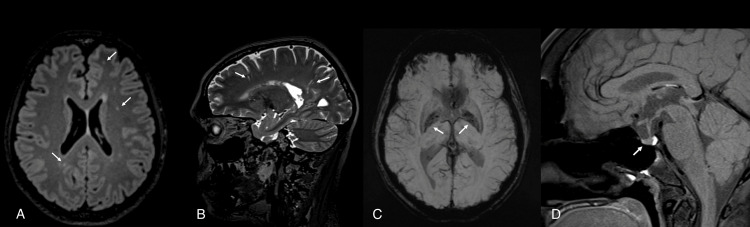
Brain MRI of Patient 1 (A) Multiple and bilateral faint FLAIR/T2 hyperintensities predominantly involving the frontal and parietal lobes; (B) sagittal T2 image showing multiple prominent perivascular spaces; (C) susceptibility-weighted imaging sequences showing mild to moderately increased mineralization/iron deposition in the globus pallidus and putamen for the patient's age; (D) the anterior pituitary measures 3.3 mm in height and is small for the patient's age.

A brain MRI showed multiple and bilateral hyperintensities predominantly involving the frontal and parietal lobes as well as a small-sized pituitary gland with evidence of iron deposition in basal ganglia (Figure 1). A 12-lead ECG showed no abnormality. She had a normal 46XX karyotype. Abdominal ultrasonography revealed bilaterally streaked ovaries with a rudimentary uterus.

Patient 2

A 43-year-old Saudi male was called to attend the clinic after the elucidation of family history and confirmation of the diagnosis of WSS in his sister. The thorough evaluation revealed a history of delayed puberty and delayed secondary sexual characteristics which were never evaluated or treated and he dropped out of school during the higher secondary stage. He had sensorineural deafness since childhood and was diagnosed with T2DM around 21 years ago, requiring treatment with metformin 500 mg TID with sitagliptin 100 mg once daily. He never had hypothyroidism.

**Figure 2:**
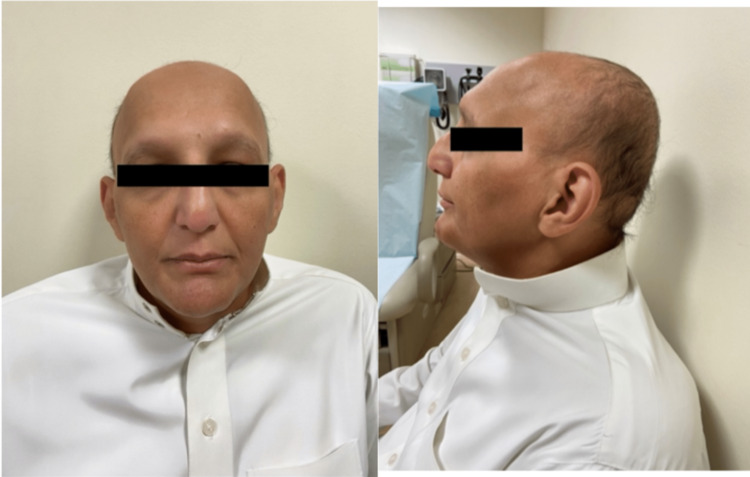
Picture of Patient 2 showing frontal and temporal alopecia, sparse eyebrows and eyelashes, widely spaced eyeballs, wasting of facial and temporal muscles, and a prominent nasal bridge

Examination showed frontal and temporal hair loss, sparse eyebrows and eyelashes, widely spaced eyeballs, and a prominent nasal bridge. In addition, wasting of the facial and temporal muscles was observed as well (Figure 2). 

The results of the rest of the clinical examination were within normal limits. His hormonal workup revealed low IGF-1, low total testosterone, and low free testosterone index with inappropriately normal FSH and LH. His pituitary profile along with adrenal and thyroid functions showed no other abnormalities. A high HbA1C along with low Vitamin D was also observed. A 12-lead ECG was within normal limits.

**Figure 3:**
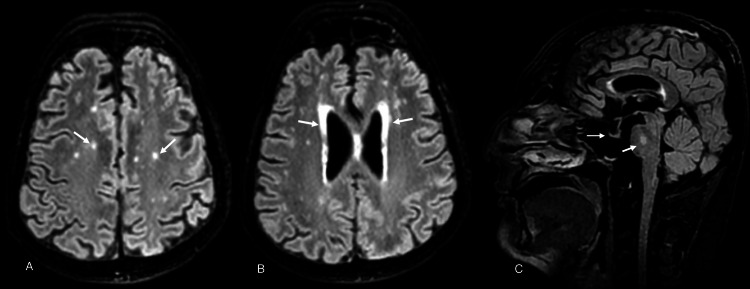
Brain MRI of Patient 2 (A) Multiple and bilateral subcortical FLAIR hyperintensities; (B) confluent bilateral periventricular white matter T2/FLAIR hyperintensities; (C) shallow sella turcica with a small pituitary gland (thin arrow) with central pontine FLAIR hyperintensity (thick arrow).

Brain MRI revealed multiple deep and subcortical discrete foci and confluent bilateral periventricular white matter T2/FLAIR hyperintensity. Exaggerated susceptibility weighted imaging (SWI) sequences hypointensities were seen involving the globus pallidus, red nucleus, and substantia nigra, suggestive of age-inappropriate iron deposition. Shallow sella turcica and a small pituitary gland, along with an abnormal craniofacial ratio with a relatively small cranial cavity were also observed (Figure 3). The patient has been advised to do semen analysis but he refused to do the same.

Patient 3

**Figure 4:**
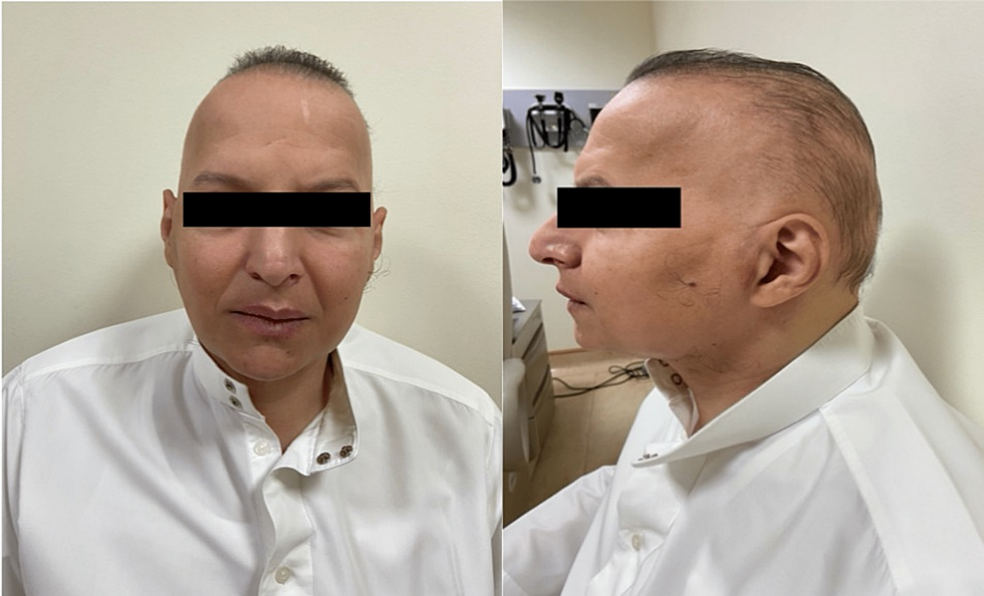
Picture of Patient 3 showing frontal and temporal alopecia, wasting of facial and temporal muscles, and a long face

The third patient is a 41-year-old Saudi male with a history of T2DM for 19 years. He had a similar clinical history to his elder brother. On clinical examination, similar findings to his elder brother were found such as a pattern of hair loss as well as hearing impairment (Figure 4). However, besides all this, he was having slowly progressive dystonia and difficulty in walking with gait instability for the last 28 years. He also had a history of decreased libido. The hormonal panel revealed intact thyroid and adrenal axis with hypogonadotropic hypogonadism. Low IGF-1, inadequate vitamin D, and a high HbA1C were other notable findings along with a normal 12-lead ECG.

**Figure 5:**
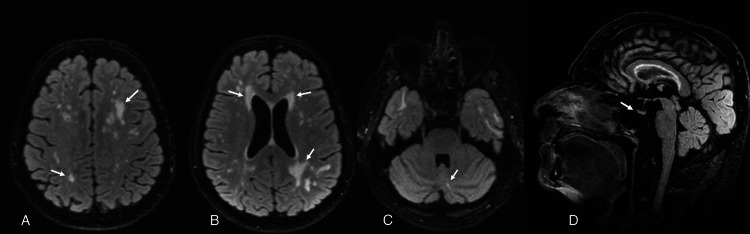
Brain MRI of Patient 3 (A) Axial FLAIR image showing bilateral frontoparietal hyperintensities sparing the U-fibers; (B) axial FLAIR images showing periventricular and deep white matter hyperintensities; (C) axial FLAIR image showing tiny left cerebellar white matter hyperintensity; (D) sagittal FLAIR image demonstrating small pituitary gland.

The brain MRI showed evidence of subacute lacunar infarcts in the right frontal and parietal lobe along with diffuse mutual periventricular and deep white matter high T2WI/FLAIR intensity foci mainly in the frontoparietal region. Features of iron deposition were observed in bilateral lentiform nuclei and a small flattened pituitary gland was seen within the sella (Figure 5). The patient refused to get a semen analysis performed.

We have summarized in Table 1 the clinical features, phenotypes, and genotypes of the patients.

**Table 1:** Clinical features, phenotypes, and genotypes of the patients TSH: thyroid-stimulating hormone; FT: free thyroxine; IGF: insulin-like growth factor; LH: luteinizing hormone; FSH: follicle-stimulating hormone; SHBG: sex hormone binding globulin; ACTH: adrenocorticotropic hormone; GH: growth hormone; PTH: parathyroid hormone

Clinical Features		Patient 1	Patient 2	Patient 3
Age at present		23 years/F	43 years/M	41 years/M
Age at the time of diagnosis		15 years	42 years	40 years
Anthropometry at presentation	Weight	54.8 Kg	77.9 Kg	78.00 Kg
	Height	151.3 cm	184.5 cm	173.0 cm
	BMI	24.03	23.6	26.00
Delayed puberty / secondary sexual characteristics		Yes	Yes	Yes
Hypogonadism		Yes	Yes	Yes
Infertility		Yes	Yes	Yes
Diabetes mellitus		Yes, for 7 years.	Yes, for 21 years.	Yes, for 19 years
Hypothyroidism		Yes	No	No
Loss of hair present in	Eyelash	Yes	Yes, partial	Yes, partial
	Eyebrows	Yes	Yes, partial	Yes, partial
	Scalp	Yes	Yes	Yes
	Face	Yes	Yes	Yes
Facial dysmorphism	Long triangular face	Yes	Yes	Yes
	Widely spaced eyeballs	Yes	Yes	Yes
	Keratoconus	Absent	Absent	Absent
	Prominent nasal bridge	Yes	Yes	Yes
	Progeroid facial skin	Not present	Not present.	Not present
Dental Loss: partial/complete		Not present	Not present.	Not present
Neurological features	Dystonia	Absent	Absent	Present
	Dysarthria	Absent	Absent	Absent
	Dysphagia	Absent	Absent	Absent
	Seizures	Absent	Absent	Absent
	Tremors	Absent	Absent	Absent
	Peripheral neuropathy	Absent	Absent	Absent
	Intellectual disability	Yes.	Yes	Yes
	Sensori-neuronal hearing loss	Absent	Present	Present
	Dystonic scoliosis	Absent	Absent	Absent
	Gait difficulty	Absent	Absent	Yes
Investigations	TSH (0.27 - 4.2 m IU/L)	12.9	4.31	2.04
	FT4 ( 9.0 – 19.0 pmol/L)	14.1	11.6	9.6
	Anti-thyroid antibody	Negative	Negative	Negative
	IGF-1 (nmol/L)	17.4 (23.8 to 126.4)	12.8 (14.2-36.9)	11.2 ( 13.8 – 38.4)
	IGF BP-3 (mg/L)	4.5 (2.2 to 7.8)	Not done	Not done
	LH (1.8 – 11.8 IU/L)	19.22	5.9	3.1
	FSH ( 3.03-8.08 IU/L)	39.13	5.5	1.5
	T. Testosterone (nmol/L)	Not done	7.88 (8.6-29)	6.5 (8.6-29)
	Free testosterone Index (%)	Not done	30.4 (35-93)	63.7 (35-93)
	SHBG ( nmol/L)	Not done	25.9 (18.3 – 54)	10.2 (18.3-54.0)
	Estradiol, E2 (pmol/L)	25.63	Not done	Not done
	Progesterone (nmol/L)	0.462	Not done	Not done
	Semen analysis	Not applicable	Refused	Refused
	ACTH (0.0-13.3 pmol/L)	8.6	4.0	8.7
	Cortisol AM (nmol/L)	310.7	634	526
	Serum cortisol (short synacthen test) nmol/L	310.7, 514.2, 589.7 at 00, 30 & 60 minutes.	Not done	Not done
	Prolactin (72-511 m IU/L)	145.1	86	Not done
	GH random (0.0 – 26 m IU/L	1.2	Not done	Not done
	GH stimulation test – clonidine Test	GH deficiency confirmed	Not done	Not done
	PTH ( 1.6 – 6.9 pmol/L)	8.55	7.75	9.85
	Celiac profile	Negative	Negative	Not Done
	HbA1C	8.9%	8.1%	8.9%
	Vitamin D (75 – 350 nmol/L)	Less than 10.0	27.8	15.1
ECG: 12-lead		Within normal limits	Within normal limits	Within normal limits
MRI - Brain	Iron deposition in basal ganglia	Present	Present	Present
	Empty sella - partial/complete	Not present	Not present	Not present
	Pituitary gland size	Small	Small	Small
	T2/FLAIR hyperintense white matter signal abnormality	Present	Present	Present
Genetic study	DCAF17 mutation	Homozygous frame shift deletion	Homozygous frame shift deletion	Homozygous frame shift deletion
	Chromosomal analysis	46 XX	Not done	Not done

## Discussion

The family of three siblings described here presented with facial dysmorphism, alopecia, subnormal intellectual levels, T2DM, and hypogonadism. These presentations are in line with those named by Woodhouse and Sakati in 1983 in highly inbred families of Saudi Arabian origin [1]. Hitherto 72 patients from 29 families have been reported in the literature [7]. Since 1983, when this syndrome was first reported, seven Saudi Arabian families have reported this syndrome and the mutation associated with it [5, 7]. Our case report adds to this tally. Apart from the confirmation of the founder effect, the autosomal homozygous nature of mutation and parental consanguinity have been established in all the cases described earlier [5]. The current report presents no exception to that fact.

Alopecia, predominantly of fronto-temporo-parietal distribution along with variable loss of hair in eyebrows, eyelashes, and general body hairs was described invariably in all the cases. Facial hypertelorism, prominent nasal bridge, and long triangular facial features together form the typical facial dysmorphism associated with this syndrome [7].

Hypogonadism is almost always present and is usually the reason for females to seek medical consultation during adolescence when they often have delayed puberty and amenorrhea; early Tanner staging and incomplete or lack of secondary sexual characteristics can be found during examination. Hypogonadotropic hypogonadism, high FSH and LH, as well as reduced or absent estradiol due to the lack of functional ovarian tissues are typically observed in females affected with WSS [1, 8]. Imaging studies of the female genital tract are helpful in confirming this finding, as seen in the proband here who had bilaterally streaked ovaries with a rudimentary uterus on pelvic ultrasonography.

Though male patients of WSS usually present with inadequate testosterone and little or inappropriately normal gonadotrophins suggestive of hypogonadotropic hypogonadism, the hypogonadism can be of central or mixed origin. They often have normal or slightly reduced testicular volume and cryptorchidism is seen infrequently, while semen analysis often shows azoospermia. There is no conclusive explanation for the observed difference in hypogonadal pattern between male and female patients of WSS. While the loss of functioning ovarian tissue and oocytes with almost no estrogen production in females and damage to testes with azoospermia in males shows a gonadal process that is peripheral, the lack of rise or suboptimal increase in gonadotrophins suggests a decreased hypothalamic-pituitary responsiveness, an important process which is central in origin. This observation has generated a hypothesis that this different hormonal profile is due to varying degrees of gonadal damage in them and not due to different mechanisms occurring simultaneously with little hypothalamic-pituitary responsiveness [1, 7, 10]. Both the male siblings in our report have hypogonadotropic hypogonadism and failed to have offspring; though they probably have azoospermia, they refused to get a semen test done to confirm this diagnosis.

Although initial case reports have not mentioned the IGF-1 level, rather only reporting an antibody level of growth hormone, the low IGF-1 level is invariably present, a finding seen and reported consistently since it was first noticed [11]. Again, multiple hypotheses have been put forward in the absence of clear pathophysiologic mechanisms of low IGF-1 status. Though a combination of low IGF-1 with normal GH levels suggests a central defect in the hypothalamic-Pituitary axis, low IGFBP-3 level, often a concurrent finding shows an abnormality in actions of GH on hepatocytes which can be due to insufficient serum levels of GH or GH receptor resistance as it is known that hepatocyte GH level regulates the secretion of IGFBP-3. Components of WSS are also present in other congenital disorders such as Laron syndrome where there is resistance to GH action due to a receptor defect or signal transduction; mental retardation, delayed puberty and temporal hair loss are also present [12].

GH resistance is also present in Noonan syndrome, along with intellectual disability and skin abnormality [13]. Lastly, considering the fact that low serum levels of steroids interfere with IGF-1 synthesis, hypogonadism may itself be the reason behind low IGF-1 levels. IGF-1 levels being lower in females compared to males has long been because of higher estrogen levels in females [14]. All three events in this report had a low level of serum IGF-1, and the proband had a low level of growth hormone too, which was confirmed with the clonidine test for GH stimulation.

T2DM has been reported in the literature in around two-thirds of the cases and the prevalence reaches around 96% with the age above 25 years [7]. The mechanism of the onset is not known completely and the lack of autoimmunity is most likely related to other gonadal dysgeneses as T2DM forms part of Klinefelter, Prader-Willi, and Bardet-Biedl syndromes [15]. All three patients in this report had T2DM and had developed it before the age of 25 years.

They reported hypothyroidism less commonly, with primary hypothyroidism without positive anti-thyroid antibodies and normal thyroid parenchyma being the most common picture. Not to mention that it can be easily treated with levothyroxine. Our proband has hypothyroidism with negative anti-thyroid antibody and she is responding adequately to replacement therapy. Hyperprolactinemia and abnormalities of the pituitary-adrenal axis have never appeared to date and our report is consistent with this [7, 16].

Neurological manifestations usually start late, at around the age of 20 years, and include faciocervical dystonia, which may lead to scoliosis and have a progressive course resulting in choreoathetoid movements and generalized dystonia, eventually leading to postural and gait abnormalities. The prevalence increases with age such that it becomes a common finding after the age of 25 years. Intellectual subnormality of moderate-to-severe grade is also common, though intra-familial variations have often been observed [7, 17]. In our report, intellectual abnormalities were observed in all three patients, although only one patient complained of dystonia and gait disorder.

Periventricular white matter abnormalities on the brain MRI have been described in the literature, along with features suggestive of iron deposition in basal ganglia. These were seen in all three of the patients described in this case, along with a small pituitary gland [7, 16-18]. The third patient in our report also had dystonia and gait disorder. Sensor-neuronal deafness has been seen infrequently, usually starting in adolescence and two patients in our report were having it.

Cardiac manifestations are rare in WSS. Electrocardiographic abnormalities such as T-wave flattening and ST-segment prolongation were mentioned initially [1] but were reported infrequently in subsequent cases [8, 17]. They are mostly asymptomatic.

Despite being a homozygous disorder, large variability in phenotypic presentation is observed in Woodhouse-Sakati syndrome, and there is no proven correlation between phenotype and genotype. The small number of cases diagnosed so far further complicates the issue and the role of other genes in its pathogenesis as a modulating factor still needs further studies. Genetic analysis for DCAF17 establishes the diagnosis, but the knowledge about the encoded protein is limited.

Although no specific therapy is available at present, the management of this entity requires multidisciplinary care. Hypogonadism could be treated with estrogen or testosterone replacement as per protocol. Screening regularly for other endocrine disorders like T2DM and hypothyroidism will help them get timely treatment, and these disorders should be managed appropriately. Neurological dysfunctions require individualized care from the neurology team along with rehabilitation work. Fertility-related issues need to be addressed by assisted reproductive techniques in an in-vitro fertilization center.

## Conclusions

WSS is an extremely rare autosomal recessive condition presenting with neuroendocrine features and is due to a mutation in the DCAF17 gene. A triad of alopecia, hypogonadism, and low IGF-1 should raise suspicion during childhood. The development of bilateral deafness and intellectual deterioration during adolescence would make it more suggestive. Later, diagnoses of T2DM and dystonia would also further strengthen the clinical investigation. Genetic testing is required to confirm the diagnosis and for appropriate genetic counseling for patients and their families. No specific treatment is available at present. Considering the diversity of the symptoms, patients need to be managed in an individualized way in a multidisciplinary care team. Further research is needed to understand the pathophysiology of this condition.
